# The Effect of Tertiary Amines as Catalysts on the Ring-Opening Polymerization of Benzoxazines

**DOI:** 10.3390/polym17111431

**Published:** 2025-05-22

**Authors:** Fanghui Liu, Ximeng Wu, Kun Chen, Junbo Yao, Qichao Ran

**Affiliations:** 1 Sinopec Key Laboratory of Drilling Completion and Fracturing of Shale Oil and Gas, Beijing 102206, China; 2 College of Polymer Science and Engineering, Sichuan University, Chengdu 610065, China; 3 State Key Laboratory of Advanced Polymer Materials (Sichuan University), Chengdu 610065, China

**Keywords:** benzoxazine, tertiary amine, curing reaction, curing kinetics

## Abstract

Benzoxazines are a kind of high-performance thermosetting resin that can undergo ring-opening polymerization to generate cross-linking structures. Here, two benzoxazine monomers, bisphenol A/aniline type (BA-a) and bisphenol A/tert-butylamine type (BA-tb), were synthesized and mixed with three tertiary amine catalysts like 2-methylimidazole (2MI), 1,2-dimethylimidazole (12MI), 4-dimethylaminopyridine (DMAP). Differential scanning calorimetry (DSC) was performed to study the curing behaviors and the curing kinetics of two benzoxazine/catalyst systems. The results indicated that all amines had a catalytic effect on the polymerization of both benzoxazines, and the BA-a/catalyst systems showed relatively higher activity. In addition, Fourier transform infrared (FTIR) and nuclear magnetic resonance (NMR) spectra were procured to analyze structural changes in the benzoxazine/catalyst systems during the curing process. The presence of the catalysts promoted the progress of the ring-opening reactions and the formation of the phenolic units in the cross-linking structures, and these evolutions were more obvious for the BA-a/catalyst system than the BA-tb/catalyst system. Furthermore, a thermogravimetric analysis (TGA) was conducted to analyze the thermal stability of the cured systems. Finally, possible curing reaction mechanisms were proposed for these benzoxazine/amine systems.

## 1. Introduction

Phenolic resin has been applied widely because of their impressive thermal stability and low cost [1,2]. However, the release of the byproducts during the curing process adversely affects its development [3,4]. A new type of thermosetting resin, polybenzoxazine, seems to resolve this issue. Benzoxazine monomers, which are typically synthesized from phenols, formaldehyde, and primary amines, have good molecular design flexibility and can undergo ring-opening polymerization to form cross-linking structures. Polybenzoxazines possess desirable properties, including near-zero volumetric shrinkage [5], high mechanical strength [6], low water absorption [7], and high char yield [8]. Particularly, there is no release of the byproducts during the whole curing process, which is favorable for preparing fiber-reinforced composites [9]. However, a very high curing temperature over 200 °C is still required for the complete polymerization of benzoxazines, which significantly prevents their development [8,10].

To solve this problem, researchers have been dedicated to moderate the curing conditions of benzoxazines [11] and found that the use of the catalysts can effectively decrease their curing temperature [12,13,14]. Dunkers [9] studied the catalyzing effects of different strong and weak acids on benzoxazines and proposed that a weak acid was expected to promote the formation of iminium ion intermediates after the ring-opening reaction, while a strong acid accelerated the formation of the Mannich bridge by an electrophilic aromatic substitution on another monomer. Given the high electronegativity of both oxygen and nitrogen atoms in the oxazine ring, some researchers hypothesized that both atoms can originate the ring-opening reaction. Based on this speculation, Wang [13] investigated the catalyzing effects of certain cationic initiators and proposed three potential polymerization pathways for benzoxazines, which would lead to two possible cross-linking structures for polybenzoxazines like the phenolic structure and the N, O-acetal structure. Moreover, some researchers [15,16] have reported that the ring-opening reaction of benzoxazines catalyzed by cationic metals was mainly caused by the protonation of the oxygen atoms in the oxazine ring. Liu and co-workers [17] proposed three main steps: the ring-opening reaction through the coordination of the metal cation with the oxygen atom, the electrophilic attack, and the rearrangement. In addition, they also examined the catalyzing effects of several Lewis acids, such as lithium salts (LiI, LiClO_4_, etc.), FeCl_3_, Zn(OTf)_2_, etc., and concluded that the anions with high nucleophilicity and good group-leaving capability can promote the polymerization. In addition, more studies regarding the polymerization mechanisms of benzoxazines catalyzed by cationic initiators were conducted in recent years [18,19,20,21]. Mustafa et al. [22] concluded from TGA tests that the incorporation of B(C_6_F_5_)_3_ as a catalyst into a bisphenol A/aniline type benzoxazine (BA-a) resulted in a decrease in T_10%_ from 364 °C to 340 °C. Transition metal salts were also proven to catalyze the ring-opening reactions of benzoxazines [22,23,24]. Wang et al. [24] added Cu(OTs)_2_ and Fe(OTs)_3_ into BA-a and found that the exothermic peak temperatures (T_p_) in DSC curves decreased from 245 °C to 222 °C and 218 °C, respectively.

To the best of our knowledge, there are a few systematic reports on the catalyzing effect of bases or nucleophilic molecules on the polymerization reaction of benzoxazines. Sun et al. [25] studied the reactions between BA-a with five different primary amines, and the results suggested that these amines can promote the ring-opening reaction. For example, the addition of m-phenylenediamine and m-xylylenediamine made the T_p_ of BA-a decreased from 234 °C to 224 °C and 220 °C, respectively. Also, our research group found that the benzoxazines can react with primary amines at room temperature [26,27]. However, the reaction between benzoxazines and tertiary amines is rarely studied.

In this paper, three tertiary amines were used to catalyze the polymerization of BA-a and bisphenol A/tertbutyline-based benzoxazine (BA-bt), respectively. Their curing behaviors, curing kinetics, structural changes during the curing process, and thermal stability of the polybenzoxazines were analyzed and compared. The possible polymerization mechanisms for the benzoxazine/amine systems were proposed.

## 2. Experimental Section

### 2.1. Materials

Bisphenol-A, tert-butylamine, paraformaldehyde, 2-methylimidazole (2MI), 1,2-dimethylimidazole (12MI), 4-dimethylaminopyridine (DMAP), aniline, methylbenzene, acetone, 1,4-dioxane, dichloromethane, isopropanol, and ethyl alcohol were purchased from Chengdu Kelong Chemical Reagents Corp., located in Chengdu, China. Trichloromethane was purchased from Chengdu Changlian Chemical Reagents Corp., also located in Chengdu, China. All the chemicals were used as received.

### 2.2. Synthesis of Benzoxazine Monomers

The synthesis processes of BA-a and BA-bt are shown in Supplementary Materials, and their structural characterization are given in Figures S1 and S2.

### 2.3. Preparation of Benzoxazine/Amine Systems

**Figure 1:**
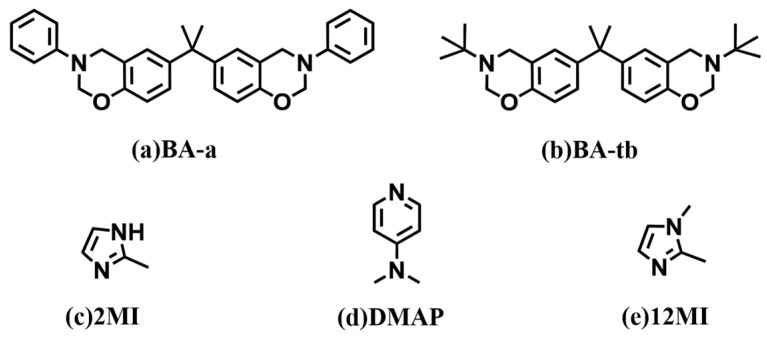
Chemical structures of benzoxazines and amine catalysts.

Three amines, 2MI, 12MI, and DMAP, were selected as catalysts for the polymerization reactions of the benzoxazines. Their molecular structures are shown in Figure 1. BA-a was dissolved in acetone and mixed with 2 mol% of amines at room temperature, and, then, the solvent was removed from vacuum oven at 30 °C. These mixtures were named BA-a/2MI, BA-a/12MI, BA-a/DMAP, respectively. In the case of BA-tb, the preparation process was similar to that of BA-a, except for two key modifications. First, dichloromethane was used as the solvent due to its superior solubility for the reactants. Second, the concentration of the catalysts was increased from 2 mol% to 10 mol% These systems were named BA-tb/2MI, BA-tb/12MI, BA-tb/DMAP, respectively.

After that, the samples were heated at 160 °C in sealed vials in an oil bath for different times. Their cured samples were named polyBA-a/2MI, polyBA-a/12MI, polyBA-a/DMAP, polyBA-tb/2MI, polyBA-tb/12MI, and polyBA-tb/DMAP, respectively.

### 2.4. Measurements

Fourier transform infrared (FTIR) spectra were conducted on a Nicolet Magna 560 spectrometer at a resolution of 4 cm^−1^. The scanning range was from 4000 cm^−1^ to 400 cm^−1^. Insoluble samples were prepared using the KBr pellet method. The sample was mixed with KBr powder and pressed into a transparent pellet under high pressure for analysis. For monomers and low-molecular-weight oligomers that could dissolve in solvents, liquid films were prepared by casting the solution onto KBr salt plates and removing the solvent under an infrared lamp. ^1^H nuclear magnetic resonance (NMR) spectra were procured with an AV-400 (400 MHZ) using deuterated chloroform as a solvent and Tetramethylsilane (TMS) as an internal standard. Differential scanning calorimetry (DSC) was performed by using a DSC Q20 (TA Instruments, New Castle, DE, USA) with different heating rates from 30 °C to 300 °C under nitrogen atmosphere. The nitrogen flow rate was 50 mL·min^−1^, and the measurements were conducted in closed aluminum pans. Thermogravimetric (TGA) experiments were acquired on a TGA 4000 (NETZSCH, Selb, Germany) with a heating rate of 5 °C·min^−1^ from 40 °C to 400 °C under nitrogen atmosphere with 60 mL·min^−1^.

## 3. Results and Discussion

### 3.1. Curing Behaviors of Benzoxazine/Amine Systems

**Figure 2:**
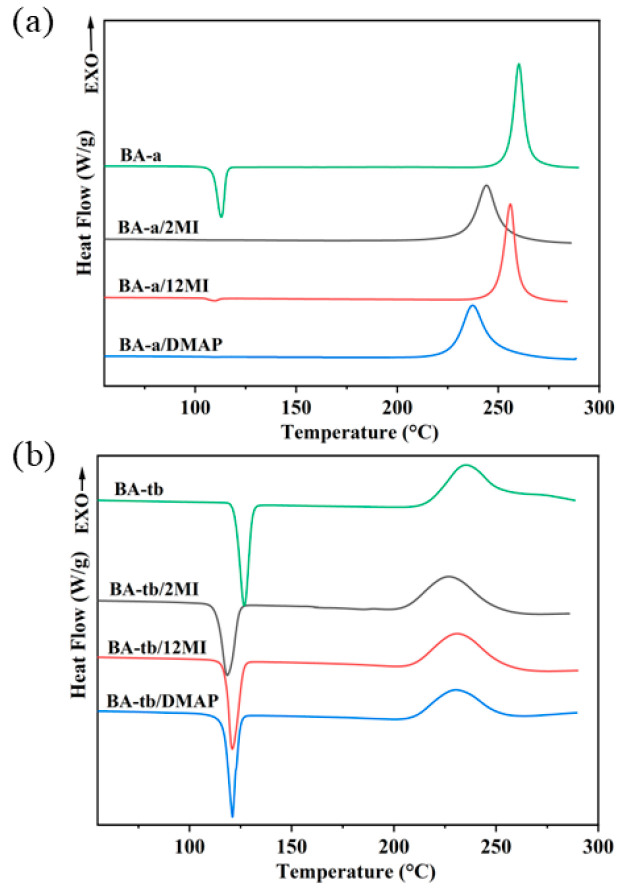
DSC curves of (**a**) the BA-a/amine system and (**b**) the BA-tb/amine system.

To investigate the effects of three tertiary amines on the curing reactions of two benzoxazine monomers, the benzoxazine/amine systems were tested by DSC. The DSC curves of the BA-a/amine system and the BA-tb/amine system are shown in Figure 2a,b, respectively, and the results are summarized in Table 1. The results showed that the T_p_ of BA-a and BA-tb were 260 °C and 236 °C, respectively. In addition, the endothermic peaks due to their melting behaviors can be observed. After the benzoxazines were blended with the catalysts in the solvents, the regularity of their crystalline regions could be disrupted, which led to changes in the melting peaks. The introduction of 2MI, 12MI, and DMAP as the catalysts resulted in obvious reductions of T_p_s to 240 °C, 242 °C, and 226 °C, respectively. It can be seen that the effect was more significant for BA-a than BA-tb, even though the additional amount of the catalysts was higher for the latter.

In contrast, as shown in Figure 2b, the DSC curves for the BA-tb/amine system showed slight reduction in both the onset temperature (T_onset_) and T_p_, suggesting a weaker response to these amines compared with the BA-a/amine system. Interestingly, the concentration of the catalysts for BA-tb is higher than the concentration of the catalysts for BA-a/amine, the effect on the curing reaction was greater for the BA-a/amine catalysts. This indicated that the catalytic activity of these amines was more effective for BA-a, which showed a significant difference in the reactivity of the two benzoxazine monomers with these tertiary amines.

In the case of BA-a, the effects of the catalysts on the T_onset_s and T_p_s followed the order: BA-a/DMAP > BA-a/2MI > BA-a/12MI, which was related to the nucleophilicity of these amines. Among them, since the presence of the hydrogen atom in the secondary amine in 2MI, the T_p_ of BA-a/2MI was lower than that of BA-a/12MI.

However, the BA-tb/amine system showed different results. Firstly, the effects of three amines on the curing behavior of the BA-tb was weaker compared with BA-a. The T_p_s of the BA-a/amine systems decreased by at least 17 °C, while the T_p_s of the BA-tb/amine systems only decreased less than 10 °C, meaning that these catalysts were more effective for the BA-a. This is the reason why more amines with 10 mol% were used for BA-tb, but only 2 mol% of the amines were used for BA-a in this work. Secondly, the effects on the T_onset_s and T_p_s of the BA-tb followed a different order like BA-tb/2MI > BA-tb/DMAP ≈ BA-tb/12MI. DMAP and 12MI showed weak catalytic effects on the BA-tb, suggesting different curing mechanisms between BA-a and BA-tb. In the case of BA-tb, the tert-butyl group has strong electron donor characteristics that increases the electron density of the C atom between the O and N atoms in the oxazine ring, which makes the nucleophilic reaction of the N atom in the tertiary amines with the C atom become difficult. In addition, the steric hindrance caused by the bulky tert-butyl group also hindered the progress of the catalytic reaction. Compared with 12MI and DMAP, 2MI showed a better catalytic effect. This improvement is due to the protonation effect of the H in the secondary amine.

Furthermore, the curing kinetics of the benzoxazine/amine systems were studied by a non-isothermal DSC method. Usually, Kissinger and Ozawa methods are used for dynamic kinetic analysis, and they are given by Equations (1) and (2) [28,29,30], respectively.

(1) $$ln\left(\beta /{T_{p}}^{2}\right)=ln\left(AR/E_{a}\right)-E_{a}/RT_{p}$$

(2) $$\ln\beta =ln\left(AE_{a}/R\right)-B-1.052{(E}_{a}/RT)$$

where β = dT/dt is the heating rate, and T_p_ is the exothermic peak temperature. Based on these equations, the activation energy (E_a_) of the curing reaction can be obtained. The DSC data for BA-a and BA-tb with various catalysts at different heating rates are provided in Table S1. The DSC curves of the BA-a/amine system with the heating rates at 10, 15, and 20 °C/min are shown in Figure S3. As expected, the exothermic peak shifted to a higher temperature for all samples as the heating rate increased, which was due to the lag of the thermal effect. Then, the E_a_s can be obtained from the linear regression of ln (β/T_p_^2^) and lnβ vs. 1/T (Figures S4 and S5), respectively. Similarly, the E_a_s of the BA-tb/amine system were obtained from Figures S6–S8. These E_a_ values are listed in Table 2.

As seen in Table 2, the E_a_s of BA-a was 96.5 and 100.3 kJ mol^−1^ from the Kissinger method and the Ozawa method, respectively. After the addition of the 2MI, 12MI, and DMAP, their E_a_s decreased to 80.0, 92.1, and 73.0 kJ mol^−1^, respectively. A low E_a_ means an easy reaction. It can be found that DMAP had the best catalytic effect, and 12MI had the worst catalytic effect based on the reduction in the E_a_s. For BA-tb, similar changes can be observed. For example, 2MI showed the highest catalytic activity and made the E_a_ of BA-tb decrease from 93.6 kJ mol^−1^ to 69.1 kJ mol^−1^, while 12MI and DMAP showed relatively low activity. The changes in the E_a_s were consistent with the DSC results. Moreover, all E_a_s from the Kissinger method were lower than those from the Ozawa method, but their trends were the same.

### 3.2. Structural Changes in the Benzoxazine/Amine Systems During Polymerization

**Figure 3:**
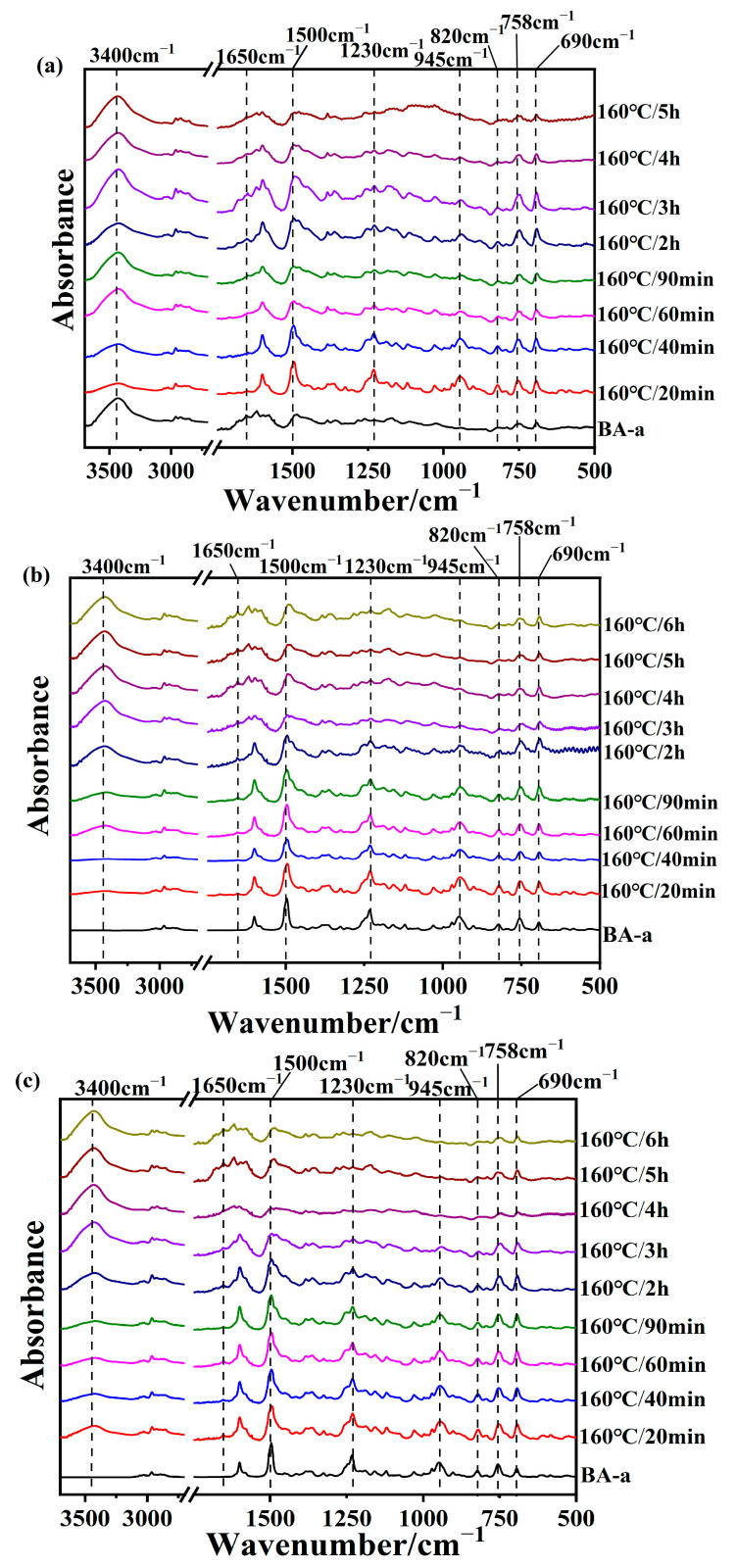
FTIR spectra of (**a**) BA-a/2MI, (**b**) BA-a/12MI, and (**c**) BA-a/DMAP cured at 160 °C.

To study structural changes in the benzoxazine monomers with different amines during the curing process, the samples were subjected to FTIR and ^1^H NMR experiments. After heating at temperatures higher than 160 °C for different durations, the obtained samples become insoluble, which in turn limits the applicability of ¹H NMR technology for analysis. Figure 3a–c show the FTIR spectra of BA-a/2MI, BA-a/12MI, and BA-a/DMAP, respectively. For the BA-a/amine systems, the band at 2960 cm^−1^ was selected as an internal reference to compare all spectra, as it was associated with the C–H bond in the methyl groups and was expected to remain. It can be seen that the reduction in the intensity of the characteristic peak at 945 cm^−1^ was associated with the ring-opening reaction of the oxazine ring for three cases [25,31]. In addition, the intensity of the peaks at 1230 cm^−1^ and 1050 cm^−1^ decreased because of the breaking of the C-O bond in the oxazine ring. Meanwhile, the intensity of the broad peak around 3400 cm^−1^ and the peak at 1283 cm^−1^ increased, which represented the generation of the phenolic hydroxyl group and the formation of the hydrogen bonds between the phenolic hydroxyl groups and the N atoms in the backbone [32]. In addition, the decreasing intensity of the peak at 1500 cm^−1^ assigned to 1,2,4-trisubstituted benzene and the increasing intensity of the peak at 1480 cm^−1^ and 820 cm^−1^ assigned to 1,2,4,6-tetrasubstitued benzene further confirmed the occurrence of the cross-linking reaction. Furthermore, there was a lag between the decrease in the intensity of the peak at 1190 cm^−1^ assigned to the C-N bond in the oxazine ring and the increase in the intensity of the peak at 1170 cm^−1^ assigned to the Mannich bridge in the cross-linking structure. In addition, the intensity of the peak at 1650 cm^−1^ assigned to the iminium ion gradually increased. This resulted from the generation of an intermediate during the ring-opening reactions of benzoxazines.

Based on the variation in the peak at 945 cm^−1^ due to the oxazine ring, it can be concluded that the ring-opening extents of three samples followed the trend: BA-a/DMAP > BA-a/2MI > BA-a/12MI. This result was also in congruity with the DSC results, and it was proven that DMAP had the highest catalytic activity. Additionally, for BA-a/2MI, the intensity of the peaks at 758 cm^−1^ and 690 cm^−1^ due to the mono-substituted aniline ring decreased, which implied that the para-position of the aniline took part in the cross-linking reaction. For BA-a/DMAP and BA-a/12MI, the intensity of these two peaks also decreased, but the extent was relatively weak, suggesting that the reaction on the para position of the aniline was not so active as BA-a/2MI.

**Figure 4:**
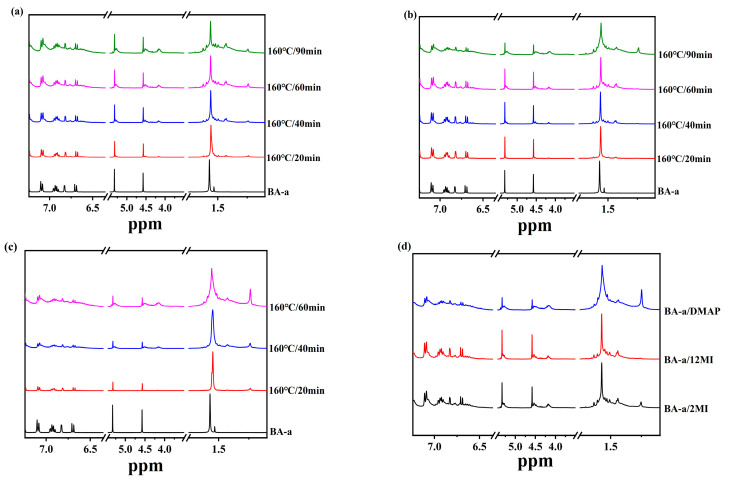
^1^H NMR spectra of (**a**) BA-a/2MI, (**b**) BA-a/12MI, (**c**) BA-a/DMAP cured at 160 °C, and (**d**) three cured samples after 160 °C for 1 h.

^1^H NMR spectra were further conducted to complement the investigation of structural change during the curing reaction. Figure 4a–c present the ^1^H NMR spectra of BA-a/2MI, BA-a/12MI, and BA-a/DMAP cured at 160 °C, respectively. The signal at 1.57 ppm due to the CH_3_ was selected as an internal standard. The bands at 4.56 ppm (Ar-CH_2_-N) and 5.32 ppm (O-CH_2_-N) due to the oxazine rings became weak gradually as the increase in the curing time, meaning the ring-opening reactions, happened. To compare the extent of the ring-opening reaction of three samples, their spectra conducted after curing at 160 °C for 1 h were compared in Figure 4d. Based on the remaining strength of the bands at 5.32 ppm and 4.56 ppm, it could be estimated that the extents of the ring-opening reaction followed the trend: BA-a/DMAP > BA-a/2MI > BA-a/12MI, which was also in accordance with the DSC and FTIR analysis results. As the curing proceeded, there were two new bands at 4.51 ppm and 4.15 ppm that appeared, which may be assigned to the O-CH_2_-N in the N, O-acetal structure and the Mannich structure in the cross-linking network.

To sum up, three BA-a/amine blends went through a similar curing process. The iminium ion was firstly generated by the ring-opening reaction of the oxazine ring followed by the formation of the Mannich bridge. Finally, the cross-linking structures with the phenolic Mannich structure and the N,O-acetone bridge structure were obtained. Among them, the BA-a/DMAP went on a relatively faster reaction than other two samples, and the para-position on the aniline ring of BA-a with 2MI could take part in the cross-linking reactions.

**Figure 5:**
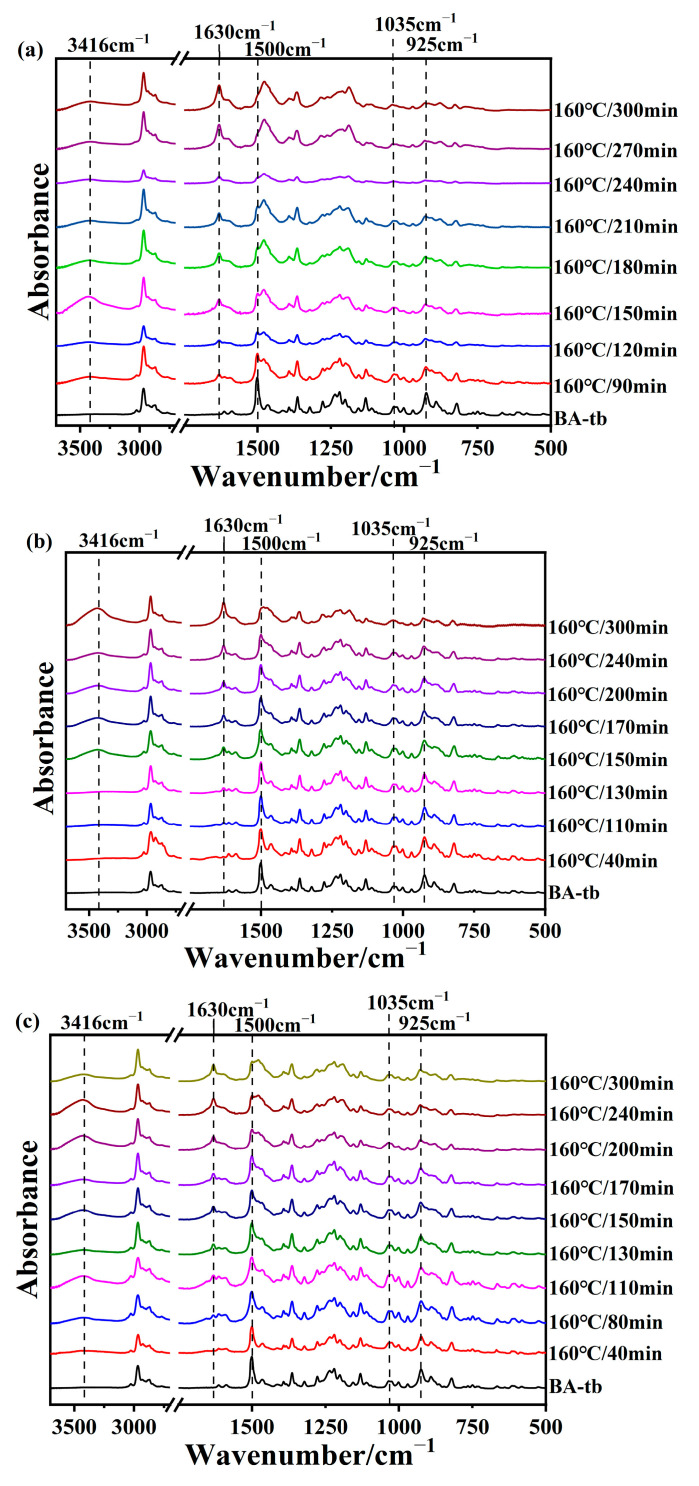
FTIR spectra of (**a**) BA-tb/2MI, (**b**) BA-tb/12MI, and (**c**) BA-tb/DMAP cured at 160 °C.

Similarly, the curing process of the BA-tb/amine system was also tracked. Figure 5a–c show the FTIR spectra of BA-a/2MI, BA-a/12MI, and BA-a/DMAP cured at 160 °C, respectively. The peak at 2972 cm^−1^ was selected as an internal standard. The intensity of the peaks at 925 cm^−1^ due to the oxazine ring and 1034 cm^−1^ due to the stretching vibration of Ar-O-C decreased, indicating that the oxazine rings opened. Also, there was an increase in the intensity of the peak at 3416 cm^−1^, suggesting the generation of the phenolic hydroxyl. Compared with the BA-a/amine systems, the decrease in the intensity of the peak at 925 cm^−1^ was relatively slow for the BA-tb/amine systems, meaning the promoting effect of these amine catalysts for BA-tb was weaker than BA-a. Among them, the catalytic effects of 12MI and DMAP seemed more weaker than 2MI. These results were consistent with the above DSC results. Moreover, the peak at 1630 cm^−1^ due to the iminium ion also appeared in the BA-tb/amine system.

**Figure 6:**
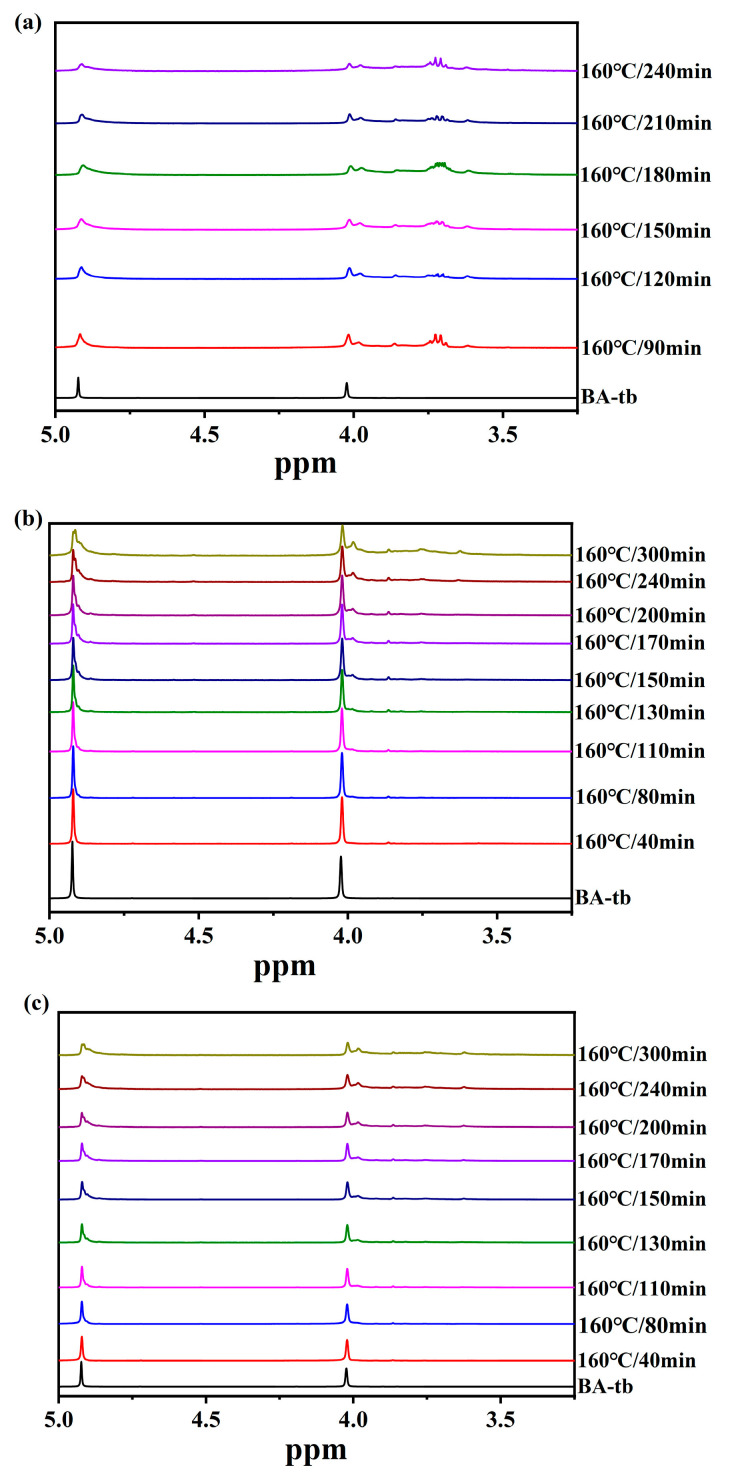
^1^H NMR spectra of (**a**) BA-tb/2MI, (**b**) BA-tb/12MI, (**c**) BA-tb/DMAP cured at 160 °C.

Furthermore, the ^1^H NMR spectra of BA-a/2MI, BA-a/12MI, and BA-a/DMAP cured at 160 °C are presented in Figure 6a–c, respectively. The characteristic bands at 4.92 ppm and 4.02 ppm due to the oxazine ring turned small for all three samples as the curing time increased, meaning the occurrence of the ring-opening reactions. It could be seen that the ^1^H NMR spectra of BA-tb/2MI was different from BA-tb/12MI and BA-tb/DMAP. The reduction extent of these bands for BA-tb/2MI was higher than BA-tb/12MI and BA-tb/DMAP, meaning faster catalytic reactions for BA-tb/2MI.

In addition, all samples after curing at 160 °C for 6 h were tested by DSC with a ramp of 10 °C min^−1^, and the DSC curves and data are shown in Figure S9 and Table S2. There were obvious exothermic peaks in all samples, meaning that these samples cannot be cured completely at this curing condition. Even so, it was meaningful because the difference among the residual enthalpies of these benzoxazine/amine systems can be studied. For cured BA-a, a residual enthalpy of 215.6 J·g^−1^ was found. After the addition of these catalysts, their residual enthalpies decreased greatly, suggesting that the catalysts promoted the polymerization of the benzoxazines. Among them, poly(BA-a)/DMAP had the lowest residual enthalpy of 135.4 J·g^−1^, meaning that DMAP had the highest catalytic effect, which agreed well with the above results. Similarly, for the BA-tb/amine system, the lowest residual enthalpy of 46.3 J·g^−1^ was found for poly(BA-tb)/2MI. In addition, the T_g_ can be obtained in these DSC curves. For the BA-a system, the addition of the catalysts increased the T_g_ greatly, which is due to more polymerization and cross-linking points. At the same time, the T_g_s of the BA-tb system were much lower. For example, poly(BA-tb) had a T_g_ below 50 °C, which was attributed to the presence of the tert-butyl group. Also, the addition of the catalysts improved the thermal resistance. The T_g_s of poly(BA-tb)/12MI and poly(BA-tb)/DMAP were increased to 63.9 °C and 70.1 °C, respectively.

### 3.3. Thermal Stability of Cured Benzoxazine/Amine Systems

**Figure 7:**
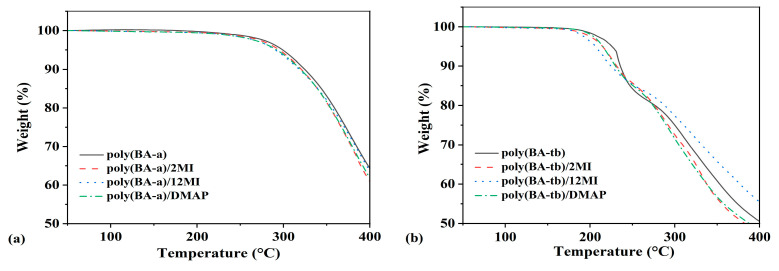
TG curves of cured (**a**) BA-a/amine system and (**b**) BA-tb/amine system.

The thermal stability of the BA-a/amine system and the BA-tb/amine system cured at 160 °C for 6 h was studied by TGA, and Table 3 summarizes the TGA results. As can be seen in Figure 7a, 5% and 10% of weight loss temperatures (T_5%_ and T_10%_) of polyBA-a decreased after adding 12MI and DMAP, while those of polyBA-a/2MI kept unchanged. This result suggested that the addition of these amine catalysts would decreased the thermal stability of polybenzoxazines. The presence of the catalysts accelerated the polymerization reactions and made the viscosity increase quickly, which may affect the normal progress of the cross-linking reactions and lead to the reduction in cross-linking density. For polyBA-a/2MI, more arylamine Mannich bridges were formed, which made the aniline ring embedded in the cross-linking structures to obtain higher thermal stability.

In addition, the cured BA-tb/catalyst systems showed similar behaviors in Figure 7b. With the addition of the catalysts, the T_5%_ and T_10%_ of polyBA-tb also decreased. It should be noted that the thermal stability of the cured BA-tb/amine systems was lower than that of the cured BA-a/amine systems. For example, the T_5%_ and T_10%_ of polyBA-tb were only 226 °C and 236 °C, which were much lower than 298 °C and 325 °C of polyBA-a. For BA-a, the para-position of the aniline took part in the cross-linking reactions, which can increase the cross-linking density. However, the tert-butyl groups in polyBA-tb were easy to go through decomposition at a high temperature. These can be proven from their DTG curves in Figure S10. The BA-a/amine system had a peak above 350 °C, while the BA-tb/amine system showed two peaks around 250 °C and 350 °C. When the weak C-N bonds were cracked, the small tert-butyl group in the BA-tb/amine system was unstable and easy to escape at a relatively low temperature.

### 3.4. Curing Mechanisms of the Benzoxazine/Amine Systems

**Scheme 1:**
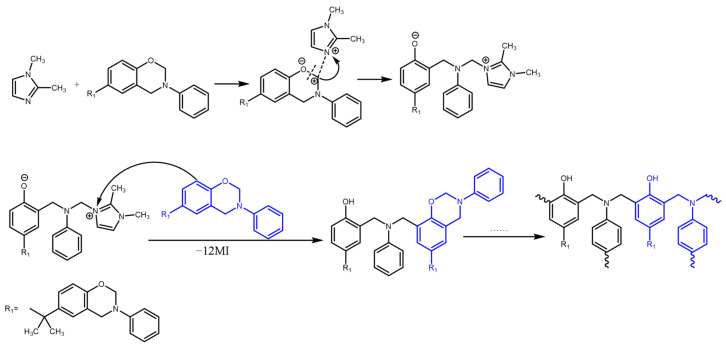
Possible curing mechanism of BA-a/12MI.

As mentioned before, the electron density of the C atom between O and N atoms in the oxazine ring is one of the factors that can affect the polymerization behaviors of BA-a and BA-tb. The possible curing mechanism of BA-tb/12MI is illustrated in Scheme S1. The electron density of this C atom is more positive for BA-a than for BA-tb, because of the weaker electron-donating effect of the aryl group, which makes the -N-CH_2_-O- more susceptible to be attacked by a nucleophile, while the stronger electron-donating effect of the tert-butyl group induces a less positive character to the same C atom in BA-tb.The reactivity of each amine varies depending on its chemical nature. In the case of 12MI, the N atoms can act as the nucleophile for the catalysis. As shown in Scheme 1, the nucleophilic reaction of the N atom in 12MI with the C atom on the oxazine ring produces an intermediate in which the positive charge is easily stabilized by the imidazole molecule. The regenerated catalyst, 12MI, could undergo a new nucleophilic reaction on the newly formed dimer and regenerate it again upon reaction with other benzoxazine monomers.

**Scheme 2:**
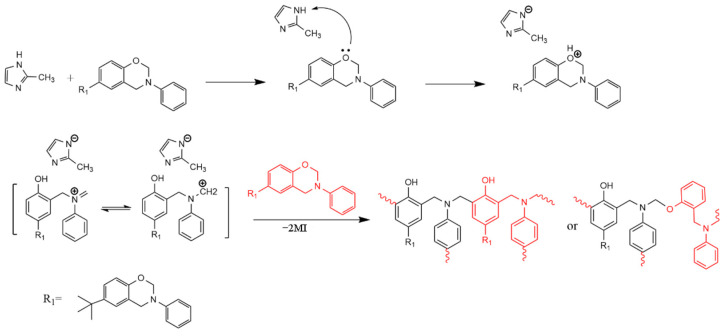
Possible curing mechanism of BA-a/2MI.

Not only can 2MI catalyze the ring-opening reaction of benzoxazine like 12MI as a nucleophile, but another catalytic mechanism is also possible due to the presence of the protons in 2MI. In this case, the polymerization reaction is initiated by the protonation of the O atom in the oxazine ring similar to other classic acids catalyzed polymerization on the benzoxazines described in the literature [33]. After losing the proton, the conjugated base imidazolate becomes a nucleophile. Two N atoms surrounding the methylene group decreases the electronic density on this C atom, which makes it more susceptible to additional nucleophilic attacks by another benzoxazine monomer. This process is followed by a proton transfer from the oligomeric structure to the imidazole. Finally, the same process is repeated sequentially to form the propagation step. The cross-linking structures dominated by phenoxy and phenolic Mannich bridges are formed, while the aniline counterparts are also involved. The proposed polymerization reaction mechanism is shown in Scheme 2.

**Scheme 3:**
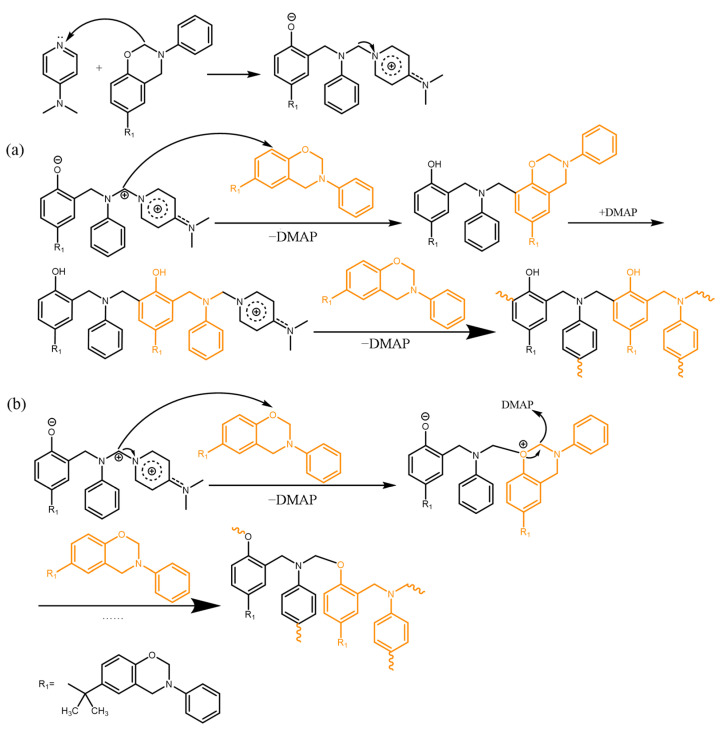
Possible curing mechanism of BA-a/DMAP with (**a**) attacking the phenoxy neighborhood and (**b**) attacking the oxygen atom.

DMAP is a strong nucleophilic reagent [34]. Therefore, it can attack the C atom on the oxazine ring and generate stable intermediates, which are efficiently stabilized by the charge delocalization with multiple resonance structures as shown in Scheme 3. This intermediate reacts with another benzoxazine monomer and regenerates the DMAP. After that, the entire polymerization reaction proceeds as 12MI.

Three tertiary amines have similar catalytic effects on BA-tb, and their possible curing mechanisms are shown in Schemes S2–S4. Unlike BA-a, the amine source of BA-tb is an aliphatic amine rather than an aromatic amine. During the curing process, the tert-butyl group in BA-t cannot be cross-linked into the network, and these suspension side groups make the polyBA-tb less thermally stable.

## 4. Conclusions

The effects of three tertiary amines (2MI, 12MI, and DMAP) on the polymerization of two benzoxazines (BA-a and BA-tb) were studied. These catalysts had higher catalytic activity for BA-a than BA-tb because of the presence of the electron-donating tert-butyl group. DMAP showed the highest catalytic activity for BA-a, while 2MI had the highest catalytic activity for BA-tb. Meanwhile, the para-position of the aniline took part in the cross-linking reaction for BA-a, and the arylamine Mannich bridge structures were formed. Moreover, the addition of these amine catalysts decreased the thermal stability of polybenzoxazines. Based on these analyses, the corresponding curing mechanisms of these benzoxazine/amine systems were proposed. These research results are meaningful for decreasing the curing temperature of benzoxazines.

## Figures and Tables

**Table 1:** DSC data of the benzoxazine/amine systems.

Sample	T_onset_/°C	T_p_/°C
BA-a	248	260
BA-a\2MI	228	244
BA-a\12MI	245	256
BA-a\DMAP	220	237
BA-tb	214	236
BA-tb\2MI	195	227
BA-tb\12MI	209	231
BA-tb\DMAP	207	231

**Table 2:** E_a_ for the BA-a and BA-tb systems based on the Kissinger and Ozawa methods.

Sample	E_a_/kJ mol^−1^	
	Kissinger	Ozawa
BA-a	96.5	100.3
BA-a/2MI	80.0	84.4
BA-a/12MI	92.1	96.1
BA-a/DMAP	73.0	77.6
BA-tb	93.6	97.2
BA-tb/2MI	69.1	73.8
BA-tb/12MI	84.9	88.9
BA-tb/DMAP	80.2	84.4

**Table 3:** TGA data of the cured products.

Sample	T_5%_/°C	T_10%_/°C
Poly(BA-a)	298	325
Poly(BA-a)/2MI	295	320
poly(BA-a)/12MI	291	318
poly(BA-a)/DMAP	284	319
Poly(BA-tb)	226	236
poly(BA-tb)/2MI	216	231
poly(BA-tb)/12MI	205	223
poly(BA-tb)/DMAP	213	231

## Data Availability

The original contributions presented in this study are included in this article/Supplementary Material. Further inquiries can be directed to the corresponding author.

## Supplementary Materials

The following supporting information can be downloaded at https://www.mdpi.com/article/10.3390/polym17111431/s1. Scheme S1. Synthesis processes of BA-a and BA-tb. Figure S1. FTIR spectra of (a) BA-a and (b) BA-tb. Figure S2. ^1^H NMR spectra of (a1) BA-a, (a2) BA-a from 7.5 to 6.5, (b1) BA-tb, (b2) BA-tb from 7.5 to 6.5. Figure S3. DCS curves of BA-a, BA-a/2MI, BA-a/12MI, BA-a/DMAP at different heating rates (10, 15, and 20 °C/min). Figure S4. Plots of $ln\left(\frac{\beta }{T_{p}^{2}}\right)$ plot $\frac{1}{T_{p}}$ by the Kissinger method for the BA-a system. Figure S5. Plots of ln(β) vs. $\frac{1}{T_{p}}$ fitted by the Ozawa method for the BA-a system. Figure S6. DCS curves of BA-tb, BA-tb/2MI, BA-tb/12MI, BA-tb/DMAP at different heating rates (10, 15, and 20 °C/min). Figure S7. Plots of $ln\left(\frac{\beta }{T_{p}^{2}}\right)$ plot $\frac{1}{T_{p}}$ by the Kissinger method for the BA-tb system. Figure S8. Plots of ln(β) vs. $\frac{1}{T_{p}}$ fitted by the Ozawa method for the BA-tb system. Table S1. DSC data of BA-a and BA-tb with different catalystsat different heating rates. Figure S9. DSC curves of cured (a) BA-a/amine system and (b) BA-tb/amine system. Table S2. Residual enthalpies and T_g_s of cured (a) BA-a/amine system and (b) BA-tb/amine system. Figure S10. DTG curves of cured (a) BA-a/amine system and (b) BA-tb/amine system. Scheme S2. Possible curing mechanism of BA-tb/12MI. Scheme S3. Possible curing mechanism of BA-tb/2MI as (a) acidic and (b) nucleophilicity. Scheme S4. Possible curing mechanism of BA-tb/DMAP with (a) attacking the phenoxy neighborhood and (b) attacking the oxygen atom.
