# Supplementary material for: The Effect of Tertiary Amines as Catalysts on the Ring-Opening Polymerization of Benzoxazines
Source: Polymers (Basel). 2025 May 22;17(11):1431. doi: 10.3390/polym17111431 (PMC12157157; doi:10.3390/polym17111431)
Supplement: Supplementary file 1 [file polymers-17-01431-s001.zip › polymers-3564842-supplementary.pdf]

Supplementary Information

The Effect of Tertiary Amines as Catalysts on the Ring-Opening Polymerization of Benzoxazines

Fanghui Liu [1], Ximeng Wu [2,3], Kun Chen [2,3], Junbo Yao [2,3] and Qichao Ran [2,3,*]

[1] Sinopec Key Laboratory of Drilling Completion and Fracturing of Shale Oil and Gas, Beijing 10083, China; liufanghui@iccas.ac.cn
[2] College of Polymer Science and Engineering, Sichuan University, Chengdu 610065, China; 2023223090033@stu.scu.edu.cn (X.W.); chen04asu@163.com (K.C.); 2024223090009@stu.scu.edu.cn (J.Y.)
[3] State Key Laboratory of Advanced Polymer Materials (Sichuan University), Chengdu 610065, China
* Correspondence: ranqichao@scu.edu.cn

Synthesis of bisphenol A/aniline type benzoxazine (BA-a)

Bisphenol-A, aniline and paraformaldehyde (suspended in a 40% aqueous solution) were used and tuluene was chosen as the solvent during synthesis. The reaction was conducted at 80 °C for 5 h. The monomer was purified by recrystallization from acetone. The general procedure for the synthesis of BA-a is shown in Scheme S1. Melting point: 113 °C (DSC). ^{1}H NMR($CDCl_3$, ppm): 7.31-6.66(m, 8H, Ar-H), 5.32(s, 4H, N-CH_2-O), 4.58(s, 4H, Ar-CH_2-O), 1.57(s, 6H, -$C(CH_3)_2$). FTIR(cm^{-1}): 2960(s, -$C(CH_3)_2$), 1600(vs, benzene), 1500(vs, 1,2,4-trisubstitued benzene), 1030, 1230(s, C-O-Ar), 945(m, oxazine ring), 758, 690(m, monosubstitued benzene).

Synthesis of bisphenol A/tert-butylamine type benzoxazine (BA-tb)

A similar procedure was followed to synthesize BA-tb. 1,4-dioxane was used as the solvent of synthesis. The reaction was carried out at 90 °C for 6 h. The solvent of the recrystallization process was isopropanol. The general procedure is summarized in Scheme S1. ^{1}H NMR($CDCl_3$, ppm): 6.97-6.62(m, 6H, Ar-H), 4.92(s, 4H, N-CH_2-O), 4.02(s, 4H, Ar-CH_2-O), 1.59(s, 6H, -C-$(CH_3)_2$), 1.19(s, 18H, Ar-C-$(CH_3)_3$). FTIR(cm^{-1}): 2972(s, -$C(CH_3)_2$), 1500(vs, 1,2,4-trisubstitued benzene), 1365, 1395(m, -C-$(CH_3)_3$),

1034, 1220(s, C-O-Ar), 925(m, oxazine ring).

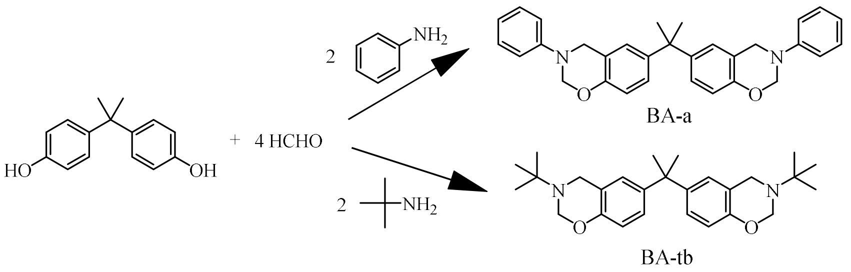

Scheme S1. Synthesis processes of BA-a and BA-tb.

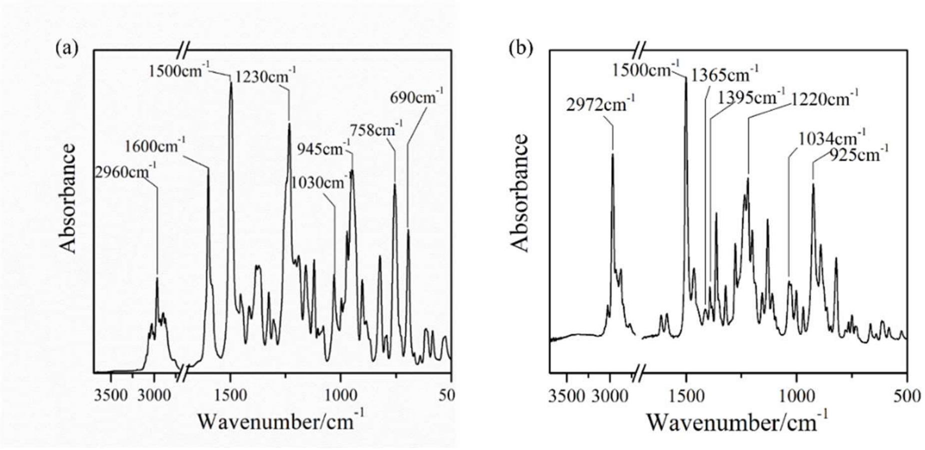

Figure S1. FTIR spectra of (a) BA-a and (b) BA-tb.

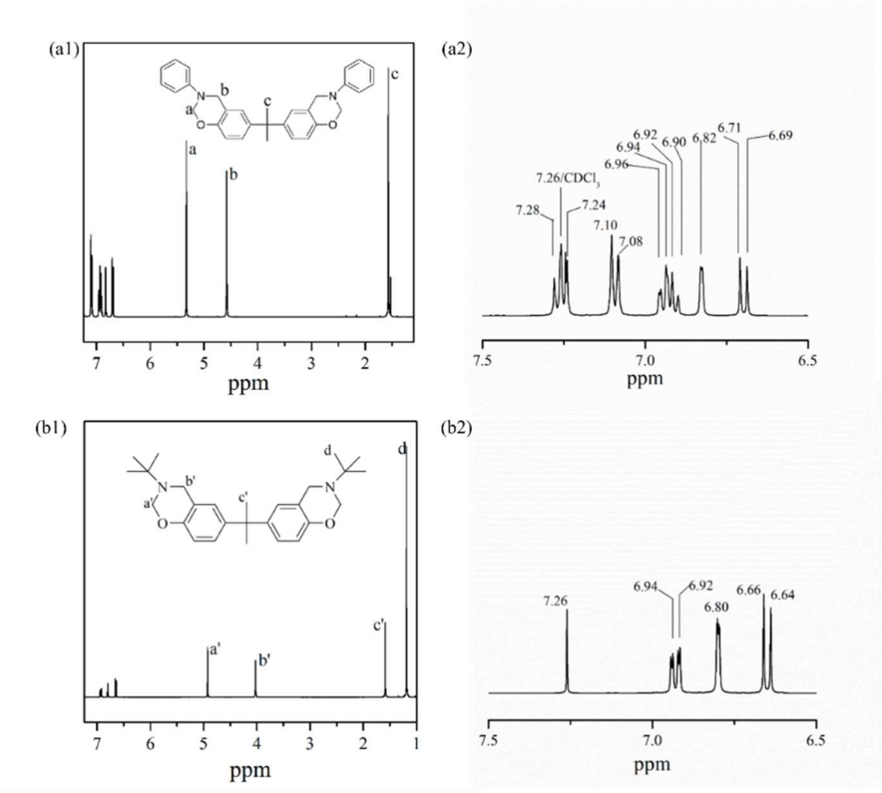

Figure S2. ^{1}H NMR spectra of (a1) BA-a, (a2) BA-a from 7.5 to 6.5, (b1) BA-tb, (b2) BA-tb from 7.5 to 6.5.

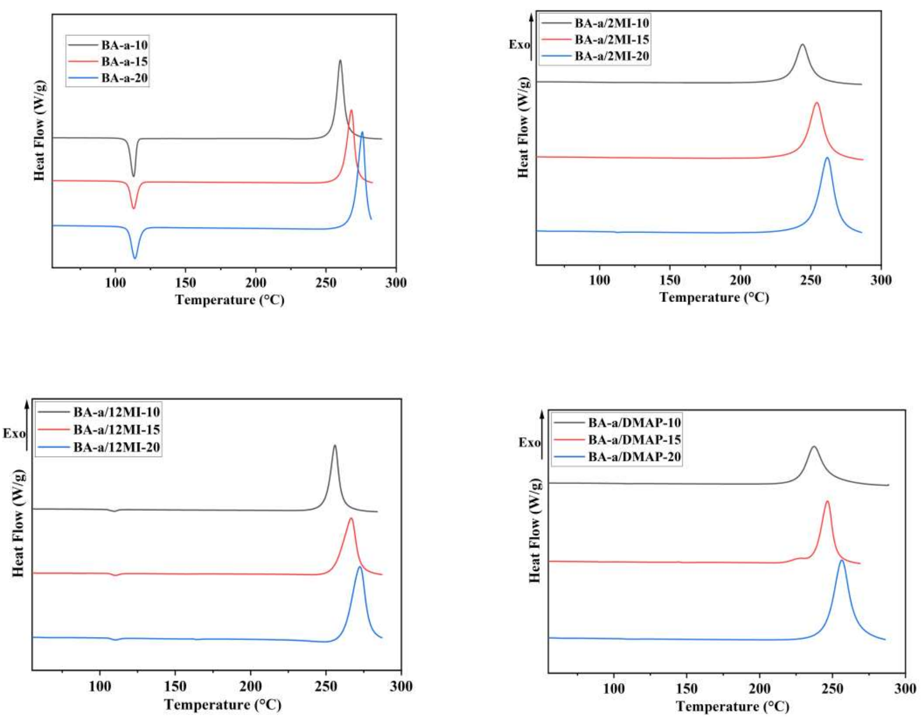

Figure S3. DCS curves of BA-a, BA-a/2MI, BA-a/12MI, BA-a/DMAP at different heating rates (10, 15, and 20 °C/min).

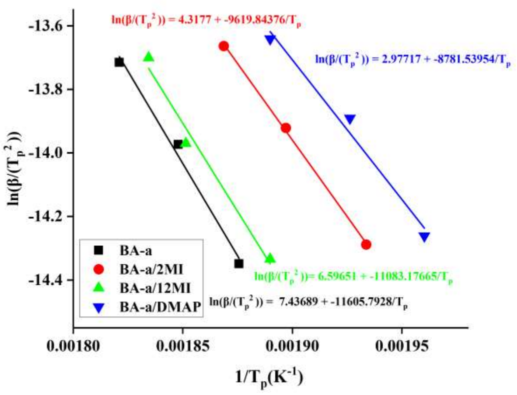

Figure S4. Plots of $ln\left(\frac{\beta}{T_p^2}\right)$ plot $\frac{1}{T_p}$ by the Kissinger method for the BA-a system.

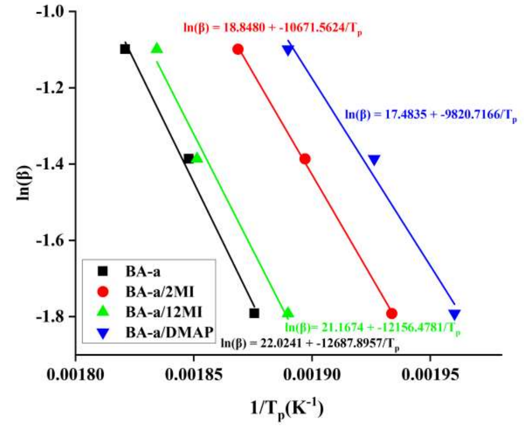

Figure S5. Plots of ln(β) vs. $\frac{1}{T_p}$ fitted by the Ozawa method for the BA-a system.

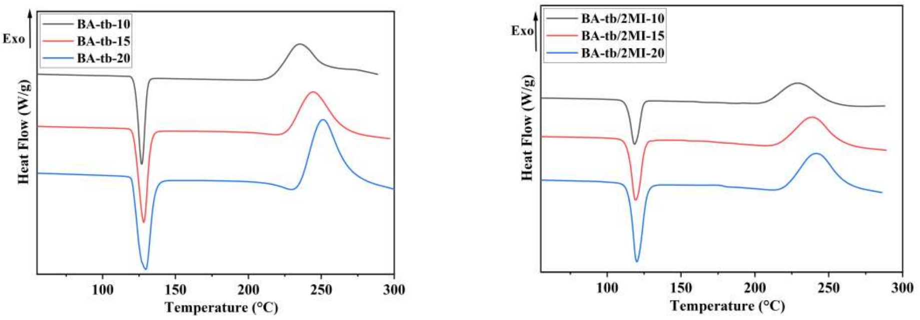

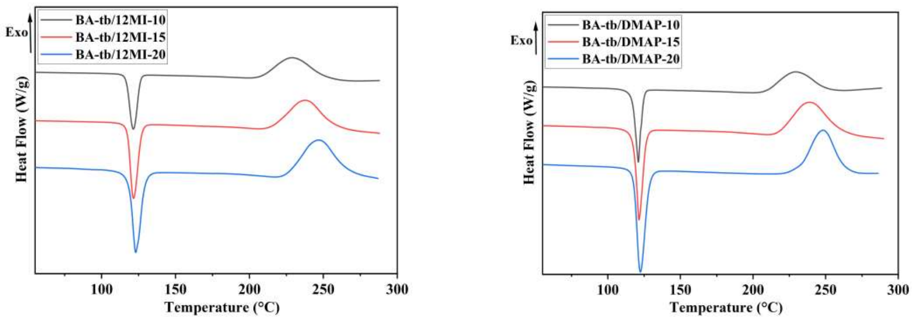

Figure S6. DCS curves of BA-tb, BA-tb/2MI, BA-tb/12MI, BA-tb/DMAP at different heating rates (10, 15, and 20 °C/min).

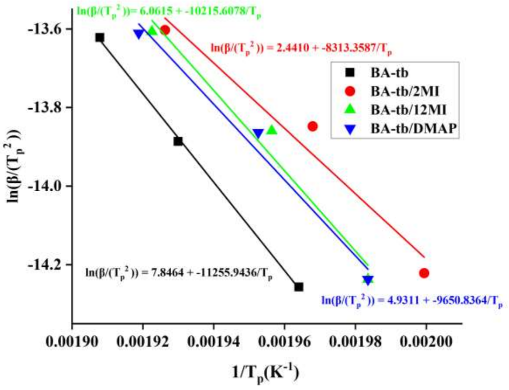

Figure S7. Plots of $ln\left(\frac{\beta}{T_p^2}\right)$ plot $\frac{1}{T_p}$ by the Kissinger method for the BA-tb system.

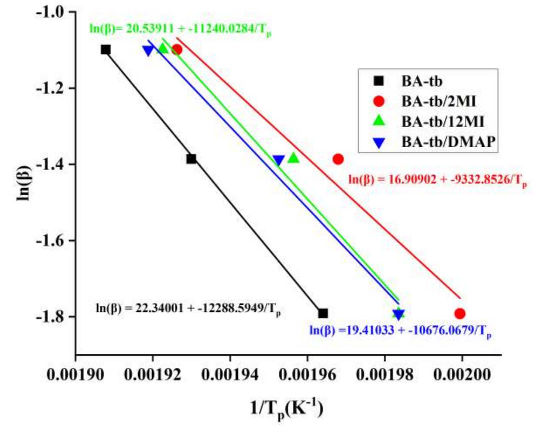

Figure S8. Plots of ln(β) vs. $\frac{1}{T_p}$ fitted by the Ozawa method for the BA-tb system.

Table S1. DSC data of BA-a and BA-tb with different catalysts at different heating rates.

Sample	β/K/min)	T_p/°C
	10	259.6
BA-a	15	268.1
	20	275.7
	10	244.1
BA-a\2MI	15	253.8
	20	261.7
	10	256.3
BA-a\12MI	15	267.0
	20	272.2
	10	236.9
BA-a\DMAP	15	246.2
	20	256.1
	10	235.8
BA-tb	15	244.9
	20	251.1
	10	227.1
BA-tb\2MI	15	235.2
	20	246.1
	10	231.3
BA-tb\12MI	15	238.1
	20	246.9
	10	231.0
BA-tb\DMAP	15	239.1
	20	248.3

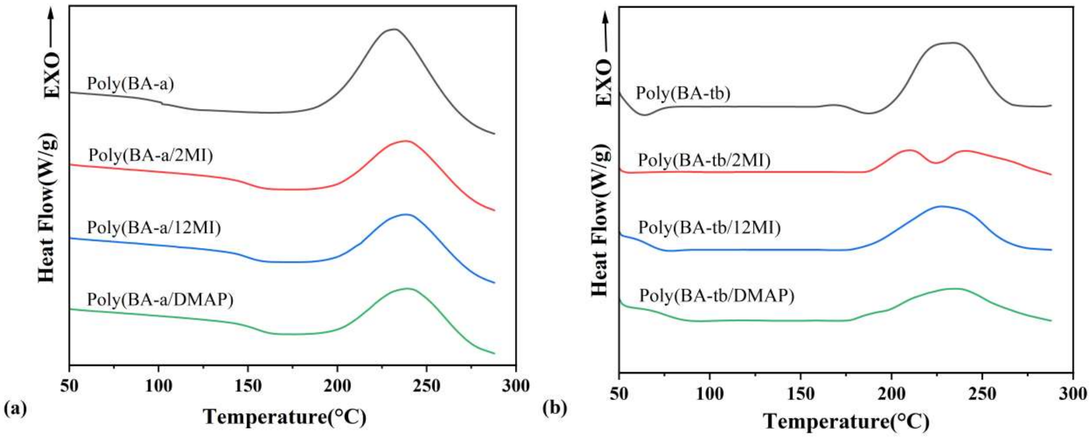

Figure S9. DSC curves of cured (a) BA-a/amine system and (b) BA-tb/amine system.

Table S2. Residual enthalpies and T_gs of cured (a) BA-a/amine system and (b) BA-tb/amine system.

**Sample**	**ΔH/J·g^{-1}**	**T_g/°C**
Poly(BA-a)	215.6	101.9
Poly(BA-a)/2MI	143.8	149.0
poly(BA-a)/12MI	141.1	149.6
poly(BA-a)/DMAP	135.4	153.8
Poly(BA-tb)	82.3	<50.0
poly(BA-tb)/2MI	46.3	<50.0
poly(BA-tb)/12MI	56.1	63.9
poly(BA-tb)/DMAP	50.8	70.1

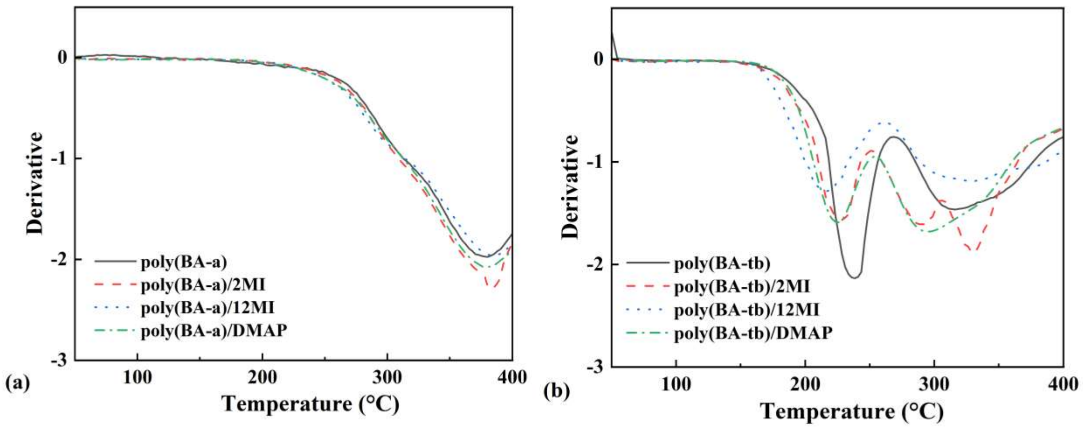

Figure S10. DTG curves of cured (a) BA-a/amine system and (b) BA-tb/amine system.

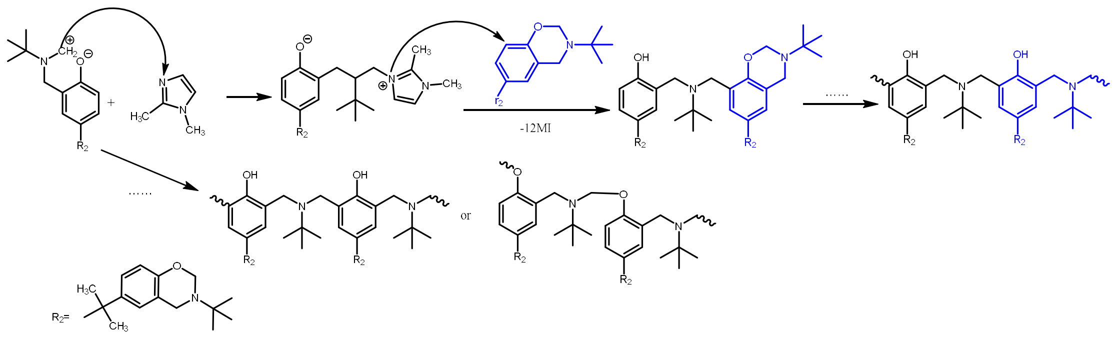

Scheme S2. Possible curing mechanism of BA-tb/12MI.

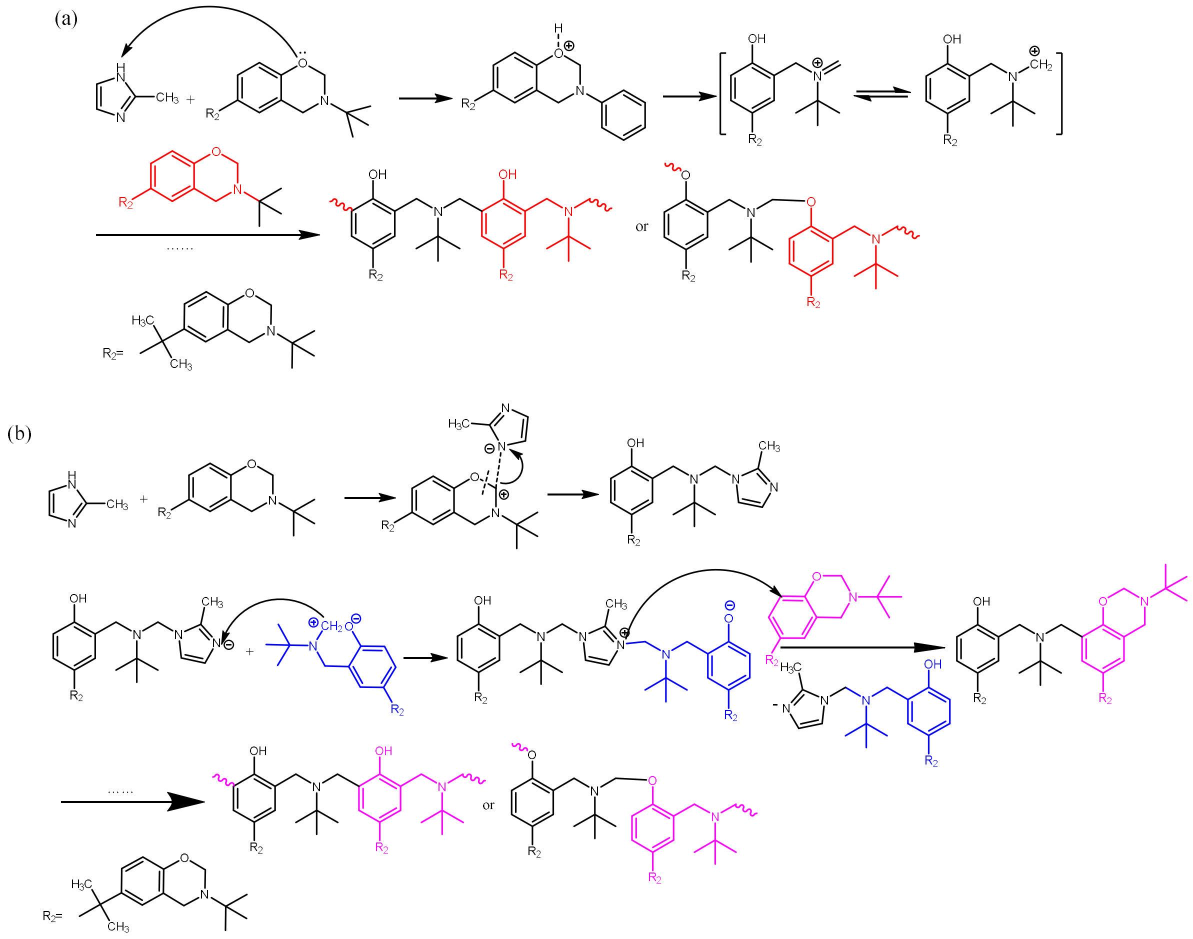

Scheme S3. Possible curing mechanism of BA-tb/2MI as (a) acidic and (b) nucleophilicity.

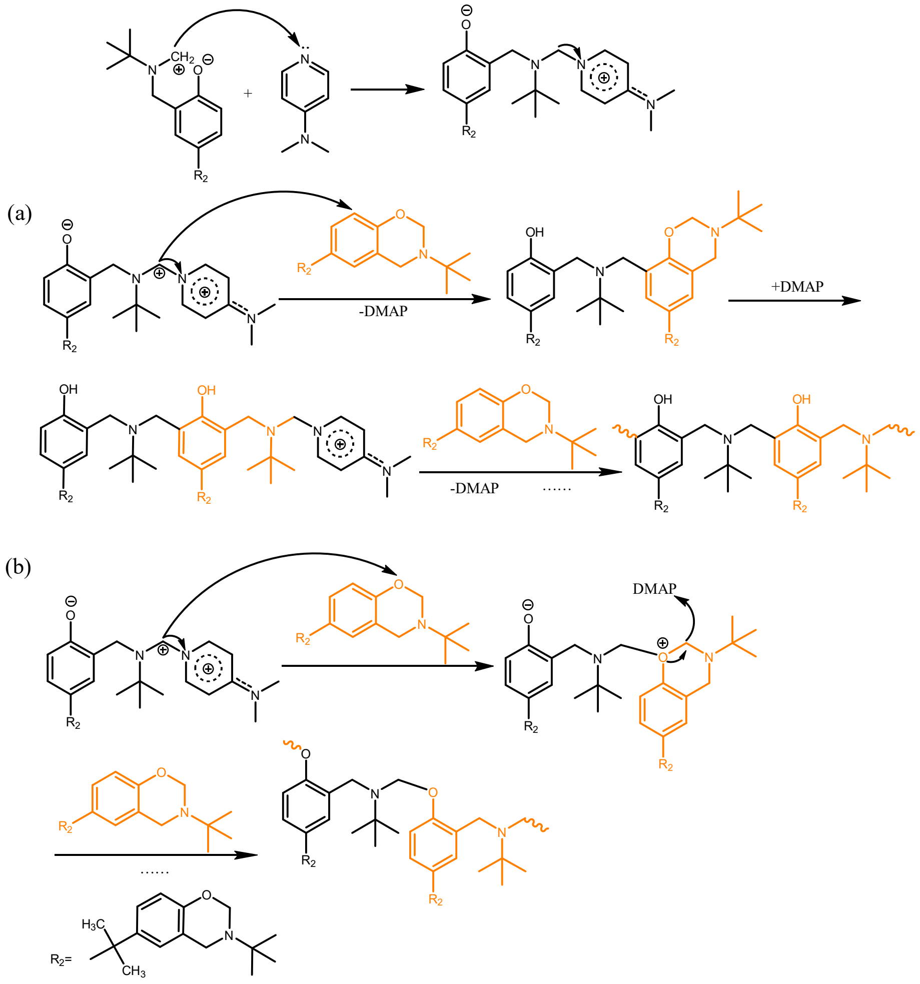

Scheme S4. Possible curing mechanism of BA-tb/DMAP with (a) attacking the phenoxy neighborhood and (b) attacking the oxygen atom.
